# Evaluation of children with ADHD on the Ball-Search Field Task

**DOI:** 10.1038/srep19664

**Published:** 2016-01-25

**Authors:** Marcos F. Rosetti, Rosa E. Ulloa, Ilse L. Vargas-Vargas, Ernesto Reyes-Zamorano, Lino Palacios-Cruz, Francisco de la Peña, Hernán Larralde, Robyn Hudson

**Affiliations:** 1 Dept. de Biología Celular y Fisiología, Instituto de Investigaciones Biomédicas, Universidad Nacional Autónoma de México, Ciudad Universitaria 04510, Mexico City; 2 Hospital Psiquiátrico Infantil Juan N. Navarro, Mexico City; 3 Instituto Nacional de Psiquiatría Ramón de la Fuente Muñiz, Mexico City; 4 Instituto de Ciencias Físicas, Universidad Nacional Autónoma de México, Cuernavaca, Mexico

## Abstract

Searching, defined for the purpose of the present study as the displacement of an individual to locate resources, is a fundamental behavior of all mobile organisms. In humans this behavior underlies many aspects of everyday life, involving cognitive processes such as sustained attention, memory and inhibition. We explored the performance of 36 treatment-free children diagnosed with attention-deficit hyperactivity disorder (ADHD) and 132 children from a control school sample on the ecologically based ball-search field task (BSFT), which required them to locate and collect golf balls in a large outdoor area. Children of both groups enjoyed the task and were motivated to participate in it. However, performance showed that ADHD-diagnosed subjects were significantly less efficient in their searching. We suggest that the BSFT provides a promising basis for developing more complex ecologically-derived tests that might help to better identify particular cognitive processes and impairments associated with ADHD.

Search behavior, defined in the context of the present study as whole body displacements made with the aim of finding a particular resource or reaching a particular goal under conditions of uncertainty, is a basic phenomenon of fundamental ecological relevance^1^,^2^. Searching is, of course, adaptive in terms of obtaining resources such as food, mates or shelter, but it can also be considered a proxy for the evaluation of the efficient functioning of a range of cognitive processes. In modern urban environments, searching is integral to many aspects of everyday life and underlies many problem-solving activities and strategies including those dealing with what to most of us are well-known if minor nuisances – where are the keys, a food item in the supermarket or when remembering the way to a favorite old shop?

Searching, following the definition provided above, involves a wide range of cognitive skills. In order to find something one must have some idea of what it looks like, avoid places that were already visited or leave places where the likelihood of finding the object is low, perhaps have an ordered or systematic strategy – try nearby places before going further and wasting valuable time and energy and possibly increasing the chance of experiencing an unpleasant or harmful event. Motivation, experience and other factors also contribute to a successful search. Certainly finding the object(s) is one criterion to end a searching process, but costs such as time and energy represent central aspects influencing the decision-making processes for an efficient search.

It is not difficult to imagine how cognitive inefficiency can be detrimental to a person’s life. For instance, attention-deficit hyperactivity disorder (ADHD) is a chronic neurodevelopmental condition characterized by atypical levels of inattention, hyperactivity and impulsivity that impact negatively on everyday functioning^3^. It is a mental health problem worldwide, with even conservative estimates suggesting it to be present in around 5% of the general population^4^. Once thought to be exclusive to childhood, it has been shown to be a lifelong disorder^5^,^6^.

The understanding of psychiatric disorders such as ADHD are substantially aided by neuropsychological tests evaluating cognitive performance^7^. However, a common problem with evaluation concerns the ecological validity of the methods used^8^,^9^,^10^. For understandable practical reasons, evaluation typically occurs in office contexts and often involves setups with an abstract framework and a goal that is foreign to the participant^11^. This is particularly problematic when assessing the cognitive performance of children, and there are still few tests that can be said to fully engage them in doing something that they might consider relevant by drawing on their everyday abilities and interests^12^.

Among some of the ingenious tests designed to meet such needs, we can mention the large-scale foraging task used by Pellicano *et al*.^13^. In this task the subject enters a room which contains a layout of light switches on the floor and must find those that turn red when activated. The test shows whether the subjects are able to understand the statistical properties of the distribution and to exploit these for more efficient searching. Also of interest is the Key Search Test^14^, a paper and pencil task in which subjects are asked to draw on a piece of paper a continuous line to simulate the searching strategy they would use to locate a lost set of keys in a field.

Here we propose an ecologically relevant test to evaluate children with and without a diagnosis of ADHD that can be applied across a wide range of ages. The test is based on previous work on searching behavior in natural and semi-natural contexts^15^,^16^ and integrates cognitive processes with actual physical costs. The test, which is reminiscent of the traditional European game of the Easter egg hunt, is a ludic task that readily captures children’s interest and their willingness to engage in physical activity. It needs few instructions and can be performed practically free from researcher interference. The large dimension setting in which it is conducted, combined with detailed automated recording of children’s pathways, provides behavioral descriptions that accurately reflect the costs involved in searching (i.e., distance travelled) and the efficiency with which collections are made (collections per meter).

The aim of the present study was to compare the performance of children with ADHD, given their lower sustained attention, with an unselected control sample in a searching task involving large open spaces.

## Methods

### Participants and Locations

We tested children from 5+ to 12+ years of age, of both sexes and without evident motor disturbances. They were recruited from two settings, a children’s psychiatric hospital and a primary school. The school sample was balanced for age and gender, whereas 81% of the participants from the hospital were male.

#### Hospital

Participants (*n* = 36, mean age 8.9 yrs, SD 1.7, range = 6.6–12.8, 28 males) were outpatients at the Hospital Psiquiátrico Infantil Juan N. Navarro in Mexico City, a public hospital specialized in the diagnosis and treatment of psychiatric disorders in children and adolescents. The inclusion criteria for participation were a diagnosis of ADHD based on a structured diagnostic interview with the parents or legal guardians (Mini-International Neuropsychiatric Interview, MINI KID)^17^, a score of at least 80 IQ points on the WISC (Wechsler Intelligence Scale for Children, WISC-IV)^18^,^19^ and to have not been on ADHD medication, at least over the course of the last year. Symptom severity was assessed by parents or guardians filling out a DSM-IV checklist according to the frequency of each of the items on a Likert scale (*never, sometimes, often*). Exclusion criteria included only comorbidity with psychosis or pervasive developmental disorders.

#### School

Participants (*n* = 132, mean age 9.2 yrs, SD 1.8, range = 5.5–12.8, 68 males) were an unselected sample of pupils at an elementary school in Mexico City.

### Ethics Statement

Methods were carried out in accordance with the approved guidelines. The Ethics Committees of the Instituto de Investigaciones Biomédicas of the Universidad Nacional Autónoma de México, the Instituto Nacional de Psiquiatría Ramón de la Fuente Muñiz, México and the Hospital Psiquiátrico Infantil approved the full procedure of the current research. Informed consent was obtained from the children’s parents or guardians.

### Test Procedures

#### The BSFT

**Figure 1:**
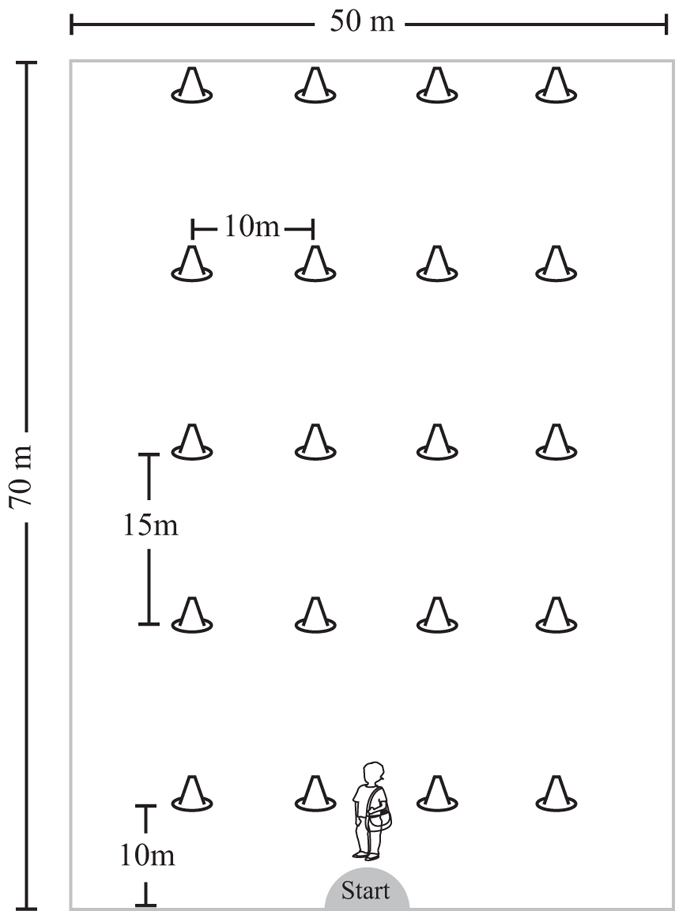
Experimental setup. Twenty orange traffic cones were distributed in a regular grid on a soccer field. A golf ball was placed under each cone and subjects were asked to collect as many balls as possible within the 8-minute test time.

The test setup consisted of a grid of 20, 30-cm high orange traffic cones arranged in 5 rows of 4 cones each on a 50 × 70 m area of a grass soccer field (Fig. 1). A golf ball was placed under each cone. Field sizes at the school and hospital were carefully measured to ensure comparability. The same set of cones and balls was used at both locations. Frequent lawn mowing was carried out on both fields.

After answering a short verbal questionnaire providing their name, age, and school class, children were brought individually to the starting point on the soccer field (Fig. 1). At the starting point the task was then briefly explained. First, the researcher pointed to the field noting the cones and asked the subjects if they were able to see all the cones. Then, the subjects were told that there was a golf ball under each one. A sample cone and ball were shown to help establish the search image. The subjects were told to try to collect all the balls, placing each in a shoulder bag, while leaving the cones as they found them (upright). Children were not told how many cones or balls there were or how much time they had to complete the task. They were given a light cloth shoulder bag and fitted with a wrist GPS (Global Positioning System; Garmin Forerunner 205, Garmin Co., USA), set to record their location every second. If the children had questions (such as “*Should I hurry?*”), these were responded to using standard pre-constructed answers that avoided specifics (“*You can do it in whichever way you want*”).

**Figure 2:**
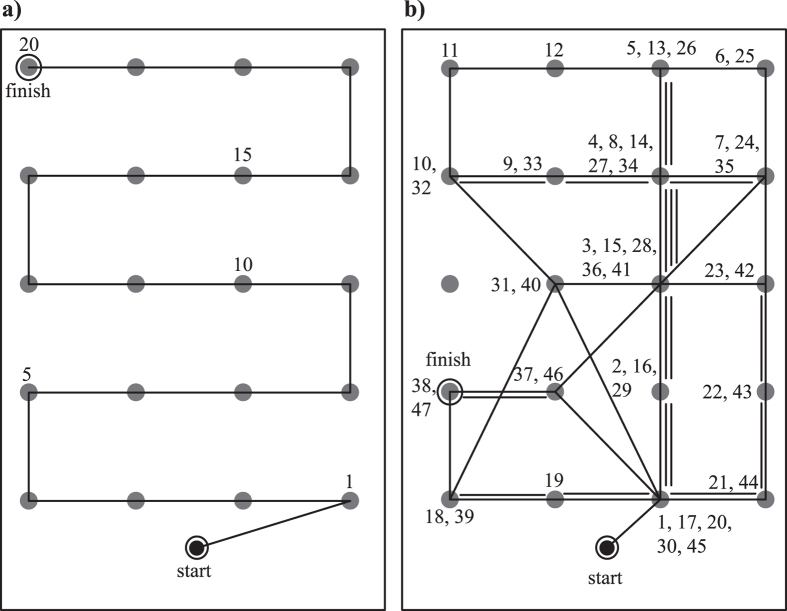
Examples of the collection sequence of two 8-year-old subjects. (**a**) A child from the school-based control group who made no mistakes and collected all balls well within the allotted 8 minutes. (**b**) A child from the hospital group with ADHD diagnosis who made many mistakes defined as returning to previously visited, “empty” cones, and who failed to collect all balls in the allotted time. Dots represent the cones, numbers give the sequence of lifting cones, multiple lines joining cones represent repeated use of the same path, and “finish” indicates when all targets had been collected or that the 8-minute test time had expired.

Two raters (PI and a research assistant) observed the child’s behavior and used a GPS-synchronized chronometer to record on a map of the cone array the time at which each cone was lifted (see Fig. 2 for examples). The experimenters ended the test when the child had collected all the balls or after 8 minutes. Based on the GPS recordings and the observers’ records, several indicators of each child’s search performance were used for analysis: 1) The number of balls collected, 2) the time taken to finish the task, 3) the distance travelled, 4) the number of mistakes made, defined as the number of returns to already visited, “empty” cones. Efficiency measures, summarizing the costs and benefits to the subjects, were calculated by the rate of collecting balls (benefit) according to the time taken per minute or to the distance travelled per meter (costs).

#### Neuropsychological evaluation

Parents or guardians of the hospital subjects completed a Behavior Rating Inventory of Executive Function (BRIEF)^20^. This rates 8 clinical scales (Inhibit, Shift, Emotional Control, Initiate, Working Memory, Plan/Organize, Organization of Materials, Monitor), used to calculate a Behavioral Regulation Index, a Metacognition Index, and a summary score, the Global Extended Composite.

Hospital subjects’ performance on the Tower of London task (ToLo)^21^, a standardized test requiring subjects to rearrange a set of balls on a set of pegs in an optimally efficient sequence, was also assessed. Performance on the ToLo is evaluated according to the number of moves (NM), the latency to the first move (L1), and the time taken to finish the test (TT). Also scored are so-called time violations (TV), defined as the number of times a subject took longer than allowed to finish a test round, and rule violations (RV) defined as the number of times a subject held more than one ball in the hand, placed a ball on the table, or attempted to place a ball onto an already full peg.

### Statistical Analysis

We used Student *t*-tests, or for nonparametric frequency data, Mann-Whitney *U* tests to compare the performance at different ages of the hospital and school groups. Pearson partial correlations, controlling for age, were performed for the hospital sample to evaluate the relationship between symptom severity, IQ and BSFT descriptors. To compare the hospital subjects’ performance on standardized neuropsychological tests against normative values we used a one-sample Student *t* test and quantified the effect size using Cohen’s *d.*

We used a two-sample test for equality of proportions^22^,^23^ to compare the percentage of hospital and school participants who collected all the balls, the percentage who collected all balls in less than half the allotted time, and the percentage who made no mistakes. To explore the possible relation between measures of absolute performance and search efficiency according to participants’ age, sex and group, we used generalized linear models, which combine continuous (here age) and categorical (here sex and group) as explanatory variables into a model. Since no interactions were found, we used a model that accounts independently for the effect of the variables involved. While a normal distribution can account for the variance in the models used for most descriptors, for the number of mistakes, a negative binomial distribution resulted in a better match due to the nature of the data^24^.

Statistical analyses and data visualization were performed using the statistical software R^25^. All tests were two-tailed and statistical significance was set at *p* < 0.05.

## Results

There was not a significant difference in age between the school and the hospital participants (Student *t*-test: *t* = 1.08, *df* = 63.92, *p* = 0.28). All subjects engaged readily in the task, and most ran rather than walked around the field. Among the children that met the inclusion criteria, none was excluded or refused to participate in the task. Even the youngest had no difficulty understanding the instructions as shown by the generally good performance at all ages (see below). No correlations were found between the performance of hospital subjects on the BSFT and symptom severity. However, positive significant correlations were found between IQ and time taken to finish the search task (*r* = −0.48) and efficiency of collections (balls per minute, *r* = 0.46).

### General Performance

A considerable number of children of both groups pursued a successful and systematic strategy, collecting all balls within the allotted time and without mistakes, that is, without returning to the same cone (see Fig. 2a for an example of the most common error-free pattern, shown by 45% of school and 26% of hospital subjects).

#### Age

**Figure 3:**
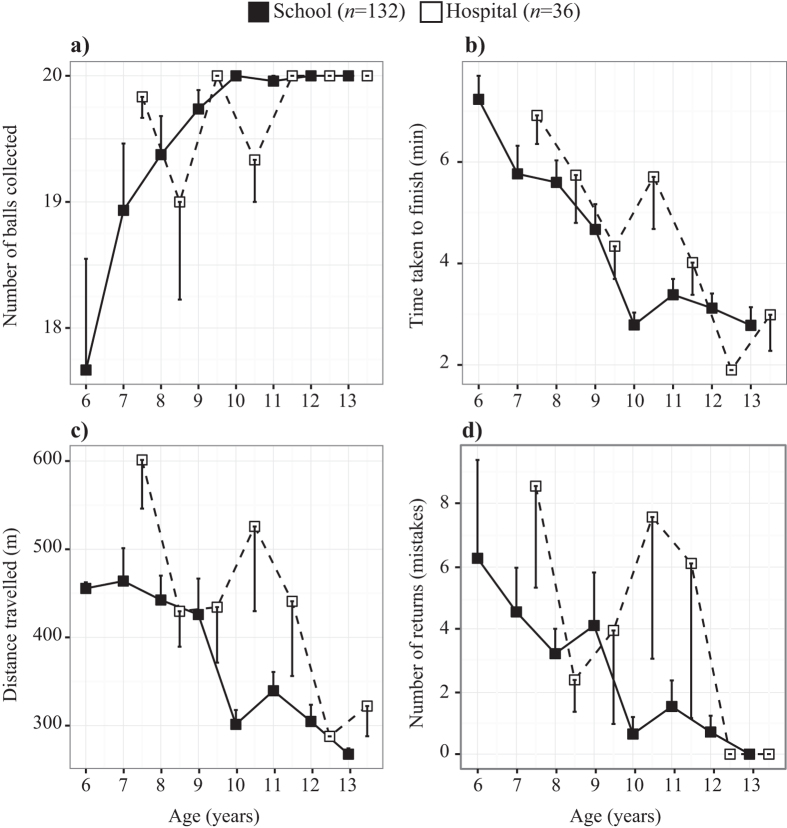
Performance with age. Improvement in general performance of subjects of the two groups with age. (**a**) Number of balls collected during the 8-minute test time (maximum score = 20). (**b**) Time taken to finish the test, that is, to collect all 20 balls or to the end of the test time. (**c**) Distance travelled during the test. (**d**) Number of mistakes defined as the number of returns to already visited “empty” cones. Open squares have been displaced slightly to the right to avoid overlap. Means and SEs are given. See text for statistical tests.

With increasing age of subjects we observed an increase in the number of balls collected, a reduction in the time taken and distance travelled to complete the task, and a reduction in the number of mistakes (Table 1, Fig. 3). By 12+ years of age, most school and hospital participants could complete the task in less than half the allotted time and without making mistakes.

#### Sex

Girls took longer to finish and travelled slightly longer distances than boys (Table 1).

#### Group

Two-sample tests for equality of proportions reported no difference between the groups in the percentage of children who completed the task by collecting all the balls (85.6% in the school, 83.3% in the hospital; *X*^*2*^ = 0.06*, df* = 1, *p* = 0.93), or in the percentage of children who finished in under 4 minutes (80.2% in the school, 72.7% in the hospital; *X*^*2*^ = 0.67, *df* = 1, *p* = 0.41). However, the percentage of hospital subjects who made one or more mistakes was significantly larger (36% and 58%, respectively; *X*^*2*^ = 4.77, *df* = 1, *p* = 0.03; see Fig. 2b for an example of a search path with many errors). Also, the average number of mistakes made per subject was larger in the hospital group, as shown by the model (Table 1).

### Efficiency

#### Age

**Figure 4:**
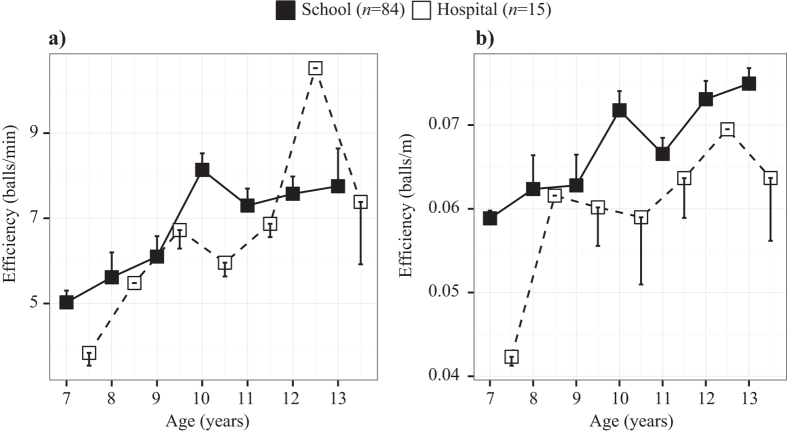
Efficiency. Improvement with age in the efficiency of subjects of the two groups in performing the task without making mistakes (returning to previously visited cones). Note the resulting reduction in sample sizes. (**a**) Time (balls collected per minute), and (**b**) distance (balls collected per meter travelled). Open squares have been displaced slightly to the right to avoid overlap. Means and SEs are given. Note that 36% of the school subjects and 58% of the hospital subjects made mistakes and so are not included in the figure. See text for statistical tests.

Searching efficiency increased with age, whether measured by the rate of collecting balls by time taken or by distance travelled (Table 2, Fig. 4).

#### Sex

Girls were less efficient than boys, as measured by the number of collections made by time and by distance travelled (Table 2).

#### Group

Children at the hospital were less efficient than the school group in the rate of collecting balls by time and by distance travelled. When only the performance of children from the two groups who did not make mistakes was evaluated (64% and 42% of the school and hospital subjects, respectively; Fig. 4), the hospital subjects were still less efficient as measured by distance travelled, but not in time taken to complete the task (Table 2).

### Children Who Made Mistakes

**Figure 5:**
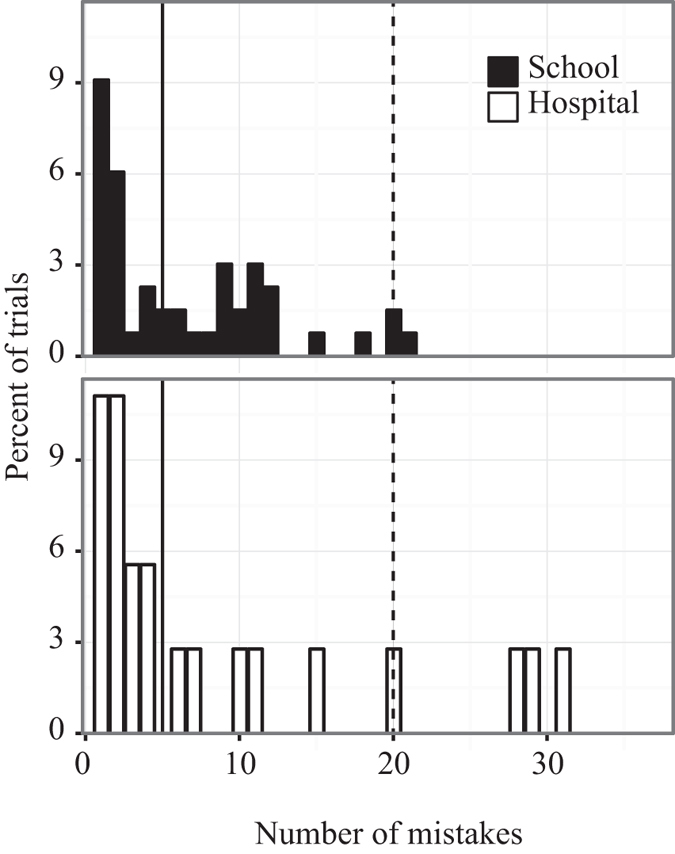
Number of mistakes. Distribution of the number of mistakes (returns to previously visited cones) made by subjects of each group as a percentage of the total number of trials (number of subjects) for each group. Values to the right of the vertical solid line indicate subjects who made ≥5 mistakes, and to the right of the vertical broken line, subjects who made ≥20 mistakes and thus the same number or more mistakes as the number of correct visits to cones resulting in collections. See text for statistical tests.

Given the generally good performance mentioned above of children of all ages from both groups, the behavior of those who made mistakes by returning to previously visited cones was particularly striking (e.g. Fig. 2b). In the school group 21 subjects (15.9%) and in the hospital group 9 subjects (25%) did so 5 or more times (Fig. 5). Indeed, in the school group 3 subjects (2.3%) and in the hospital group 4 subjects (11.1%) made as many or more returns to empty cones as they made collections (e.g. Figs 2b and 5). This resulted in generally longer search times and 14.4% of school subjects and 16.7% of hospital subjects, all of whom made mistakes, did not complete the task within the 8 minutes, although all continued energetically searching to the end of the allotted time.

### ToLo, BRIEF and the BSFT

Children at the hospital were rated as having poor executive performance by their parents or guardians. The values for all items of the BRIEF were significantly different from the population mean, with large size effects (values for Cohen’s *d* ranged from 1.6 to 2.6). Their performance on the ToLo was significantly worse only on number of rule violations (*t* = 2.19, *d* = 0.43). Despite the BSFT showing a certain capacity for discrimination between samples, and the use of commonly used tasks to evaluate some aspects of cognitive function in ADHD subjects, no correlation was found between any of the performance measures of the BSFT, ToLo and BRIEF scores or between the ToLo and BRIEF scores.

## Discussion

There is an increasingly recognized need for ecologically valid tests^8^,^9^,^10^,^11^,^12^,^26^,^27^ capable of detecting developmental changes in cognitive function and generating descriptors that differentiate between the performance of control and clinical groups in order to further our understanding of childhood neuropsychological disorders. From the results of this first study, we suggest that tasks based on searching, such as the BSFT, can provide possibilities to utilize situations that involve subjects weighing the costs and benefits in decision making processes.

### The BSFT

The task was clearly well accepted, even by the youngest children and including by the patients in an unfamiliar hospital setting. Subjects’ motivation to perform well and their response to the task as an interesting, intrinsically motivating, and readily understood game was shown by the fact that most children ran, sometimes considerable distances (Fig. 3c), and managed to collect all the balls well within the allotted time (Fig. 3a,b). Furthermore, the good performance of 5 to 6 year-olds suggests that a modified version might be useful in the evaluation of neuropsychological functioning in even younger children with ADHD, for whom there are relatively few studies^28^ (but see^29^,^30^).

#### Strategy

Most collection patterns followed a nearest neighbor strategy. The grid structure of cones seemed to promote sequentially picking up most cones along rows or columns. Although this decision making strategy can be perceived in at least part of most paths, on occasion the searcher would move diagonally or even skip a row. Many of the mistakes can be traced back to these types of decisions, which may reflect poor attentional control.

#### Age

The improvement in performance on all parameters with age and in both groups of subjects (Figs 3 and 4) suggests that the task, even in its present form, is sensitive enough to track developmental progress on decision-making processes associated with attention and memory. Among the explanations for subjects improving with age is certainly an increase in gross motor ability. Nevertheless, this cannot be the only explanation given the differences in parameters such as the distance travelled or the number of mistakes made (Figs 3 and 4). Thus, it appears that the maturation of cognitive abilities also contributed to the improvement in performance^31^.

#### Sex

The significant effect of sex on performance suggests that female subjects had less efficient search paths than male subjects. While our unselected control sample had an even proportion of female and males, the ADHD sample contained a larger percentage of male subjects given this commonly found bias in referral rates^32^. In the current study, the differences between sexes would presumably have been even larger if the proportion of sexes in the two samples had been the same.

Sex differences in performing tasks that require spatial abilities have been a long-debated topic^33^,^34^. In the present study we found that boys outperformed girls of the same age in the time needed to complete the task, and we have previously reported sex differences in searching behavior in a variety of contexts^16^,^35^. The relevance of these differences in the current task is two-fold. First, the fact that these differences appear in such a variety of scenarios (outdoors and indoor pencil-and-paper tasks) suggests that search behavior is a robust, biologically relevant activity and thus, an excellent basis for assessment. The second point is that sex should be taken into account when establishing normative values for any search-related activity.

#### Group

We observed differences in the performance of hospital and school children. Although hospital subjects appeared motivated to perform the task and to have understood the instructions, they had search pathways that were less efficient in terms of average time taken and distance travelled, and they also made more mistakes (Figs 4 and 5). This might have been due to the poor attention and memory skills often associated with ADHD^36^, although it is difficult to discard the negative effects of being tested in an unfamiliar hospital setting in contrast to the control subjects who were tested in their familiar school environment. However, this is often the case with neuropsychological tests performed in hospitals or doctor’s offices. Nevertheless, it should be noted that a considerable number of hospital subjects performed as well as control subjects, for example, by collecting all balls, mistake free, and well within the allotted time (Fig. 3).

ADHD has been associated with a variety of cognitive deficits^37^, and thus the good performance of some of the ADHD-diagnosed children could have different explanations. A first possibility is misdiagnosis or different degrees of severity. Another possibility is the inclusion of several subtypes in the sample, such as patients with *high-functioning* ADHD^38^, who show high intelligence combined with high distractibility. Yet another option is that some of the subjects, living all their life with the disorder, might have learned to compensate for associated cognitive deficits^39^. For example, although the hospital children who succeeded in collecting all of the balls without mistakes travelled greater distances (took less systematic, less optimal paths) than the correspondingly successful controls, they achieved this in the same overall time by searching with greater speed. Reasons for a small percentage of the school children performing almost as poorly as the worst-performing hospital subjects are presently unclear, but might suggest undiagnosed cognitive problems, perhaps going unnoticed by parents or teachers due to the buffered social environment of a private school. In general, clarification of the reasons for the considerable overlap in the performance of the two groups awaits the application of more discriminating versions of the BSFT.

#### Neuropsychological validity

Despite performance on the BSFT appearing to be informative about the behavioral consequences of ADHD, we found no relationships between the BSFT, ToLo and BRIEF scores. However, given that measures of cognitive abilities have been a controversial topic due to the loose definition of concepts and classifications^40^, we are cautious in drawing conclusions from these early comparisons, particularly without further exploration using larger sample sizes. As other authors have noted^41^,^42^, we appear to be still struggling for robust neuropsychological tools. Nevertheless, given the current difficulties presented by neuropsychological evaluation, the best route is to use multiple, including ecologically valid, forms of assessment to capture different aspects of cognitive performance.

In comparison with other neuropsychological tests, the BSFT requires only brief training to administer, balls and cones can be readily substituted by other targets and covers, and it can be implemented in any sufficiently large outdoor or indoor space. Also, although GPS recordings provide valuable fine-tuned information on children’s search pathways, the main search parameters can be easily registered by observers using pencil, paper and a chronometer (e.g. Fig. 2).

#### Visual search and foraging

As Mullane & Klein^43^ have pointed out, there have been several attempts to use visual search tests to identify the underlying cognitive processes involved in the attentional difficulties characterizing ADHD. The tests used for visual attention require rapid scanning of images to locate a target hidden among distractor items. They represent a highly controlled experimental situation to assess visual searching efficiency. While similarities exist between visual searching and searching that involves full body displacement, authors such as Gilchrist, North, & Hood^44^ have also remarked on the differences, particularly in relation to the energetic and cognitive demands of a search involving full body displacement. It is also the case that a search process requiring displacement will also involve visual scanning, thus becoming a good candidate to evaluate attentional difficulties, lack of inhibitory control and hypermobility in a single task.

### Implications of the Study

The wide prevalence of ADHD has led some researchers to turn to evolutionary biology for suggestions as to how some of the main symptoms – hyperactivity, inattentiveness and impulsivity – may be of advantage in ancestral environments. Williams & Taylor^45^ mention a possible social role, whereby the presence of impulsive decision makers might provide information enabling other group members to make better choices, thus improving group fitness. Shelly-Tremblay & Rosen^46^ consider how ADHD symptoms could have had a positive effect on several activities of ancestral humans, such as hunting, fighting, and wading, in the attempt to understand a psychiatric disorder as a former adaptation. And Jensen *et al*.^47^ suggest potential roles for particular symptoms – could children’s increased motor activity improve their foraging performance via a more vigorous effort to find resources? Or could children’s inattention make them poor foragers, unable to maintain a systematic strategy and returning often to previously visited depleted sites? Provisionally, our results suggest that the symptoms of ADHD do not give rise to efficient foraging behavior [cf.^13^].

In the context of such discussion we believe it can be useful to design neuropsychological tasks creating situations where children with a disorder such as ADHD can potentially excel rather than situations where they are almost certainly set to fail. In this way, we might advance our understanding regarding the contexts in which the defining symptoms of ADHD are manifested and maintained, as well as elucidate the cognitive limitations that such symptoms entail.

### Perspectives

We did not know at the start of this study how children, particularly at a young age and in a hospital setting, would respond to this task; that is, how difficult it would be for them and how well they would cooperate. The unexpected ease and enthusiasm with which most children performed the BSFT suggest that it might indeed tap into behavioral and cognitive abilities which are in tune with basic biological concerns^48^,^49^. The task, although simple in design, can very quickly become complex when the subject’s distractions result in a failure to follow a systematic strategy and in difficulties remembering already visited cones. The current setup provides a necessary baseline for future configurations, and for testing other deficits than ADHD. For example, in the BSFT targets can be re-arranged to produce more complex, challenging arrays than the regular, predictable arrangement used here. The current configuration of cones and balls uses the most basic search scenario, where the searcher is introduced to the search arena for the first time. Among the limitations of this scenario is that in everyday life people often search in familiar places and have to remember an object’s last location. Not only memory, but also experience modifies searching efficiency. Such scenarios can be readily addressed by further configurations of the BSFT and by repeated testing. For instance, it could be possible to test working memory by allowing children to hide the balls under some of the cones and retrieve them after a given time. Also, the dimensions of the search field can be possibly reduced, perhaps even to table-top or computer-screen versions, similar to those used in the Key Search Task. However, we suspect that reducing or eliminating the gross motor feedback associated with the physical demand presently required to complete the task might deprive it of an important part of its ecological validity^50^,^51^,^52^.

In conclusion, future studies using search behavior to evaluate the effects of neurodevelopmental disorders on motor and cognitive performance could include tests like BSFT for comparison with commonly used neuropsychological tests, as well as to test the effects of pharmacological treatments.

## Additional Information

**How to cite this article**: Rosetti, M. F. *et al*. Evaluation of children with ADHD on the Ball-Search Field Task. *Sci. Rep.* **6**, 19664; doi: 10.1038/srep19664 (2016).

## Figures and Tables

**Table 1:** Effect of age, group and sex on general performance descriptors.

Descriptor	Explanatory variables	Coefficient ± SE	*t*value	*p*
*Collections*	Age	0.01 ± 0.01	0.98	0.33
	Group (*Hospital vs. School)*	0.001 ± 0.02	−0.06	0.95
	Sex *(Girls vs. Boys)*	0.005 ± 0.02	−0.26	0.79
*Distance*	Age	−32.99 ± 5.6	−5.89	<*0.0001*
	Group (*Hospital vs. School)*	−46.98 ± 12.74	−3.69	<*0.001*
	Sex *(Girls vs. Boys)*	20.37 ± 10.55	1.93	0.06
*Time*	Age	−0.6 ± 0.07	−8.12	<*0.0001*
	Group (*Hospital vs. School)*	−0.42 ± 0.17	−2.48	*0.01*
	Sex *(Girls vs. Boys)*	0.39 ± 0.14	2.80	<*0.01*
*Mistakes*	Age	−0.37 ± 0.09	−3.91	<*0.0001*
	Group (*Hospital vs. School)*	−0.49 ± 0.21	−2.37	*0.02*
	Sex *(Girls vs. Boys)*	0.22 ± 0.17	1.26	0.21

Sign of the coefficients indicate the direction of the relationship.

**Table 2:** Effect of age, group and sex on collection efficiency.

Descriptor	Explanatory variables	Coefficient ± SE	*t*value	p
All Children				
*Efficiency by time*	Age	0.74 ± 0.08	8.75	*<0.0001*
	Group (*Hospital vs. School)*	0.54 ± 0.19	2.80	*<0.01*
	Sex *(Girls vs. Boys)*	−0.47 ± 0.16	−2.99	*<0.01*
*Efficiency by distance*	Age	0.004 ± 0.0006	8.03	<*0.0001*
	Group (*Hospital vs. School)*	0.005 ± 0.001	3.94	<*0.001*
	Sex *(Girls vs. Boys)*	−0.002 ± 0.001	−2.16	*0.04*
Children who did not make mistakes				
*Efficiency by time*	Age	0.48 ± 0.1	4.63	<*0.0001*
	Group (*Hospital vs. School)*	0.33 ± 0.24	1.37	0.18
	Sex *(Girls vs. Boys)*	−0.39 ± 0.18	−2.21	*0.03*
*Efficiency by distance*	Age	0.003 ± 0.0006	4.41	<*0.0001*
	Group (*Hospital vs. School)*	0.004 ± 0.001	3.02	<*0.01*
	Sex *(Girls vs. Boys)*	−0.0006 ± 0.001	−0.66	0.55

Sign of the coefficients indicate the direction of the relationship.
